# Supplementary material for: FeCu dual-single-atom catalyst promotes gradient H2O2 activation for enhanced methane oxidation to methanol
Source: Nat Commun. 2026 Mar 5;17:3526. doi: 10.1038/s41467-026-70179-8 (PMC13087317; doi:10.1038/s41467-026-70179-8)
Supplement: Supplementary file 1 — Supplementary Information [file 41467_2026_70179_MOESM1_ESM.pdf]

Supplementary Information for

FeCu Dual-Single-Atom Catalyst Promotes Gradient H_2O_2 Activation for Enhanced Methane Oxidation to Methanol

Haonan Zhang [1]†, Shuai Wang [2]†, Yang Li [1], Hongjie Qin [1], Mingwang Wang [1], Qinghai Chen [1], Boshi Zheng [1], Shuxu Zhu [1], Pengye Zhang [1], Chaoqun Gu [1], Yunyun Li [1], Qi Hua [1], Mingbo Wu [1, 3]*, Wenting Wu[1]*

[1] State Key Laboratory of Heavy Oil Processing, College of Chemistry and Chemical Engineering, Institute of New Energy, China University of Petroleum (East China), Qingdao 266580, P. R. China

[2] China energy engineering group jiangsu power design institute Co., Ltd. Jiangsu 211100, P. R. China.

[3] State Key Laboratory of Advanced Optical Polymer and Manufacturing Technology, College of Chemical Engineering, Qingdao University of Science & Technology, Qingdao 266061, P. R. China.

† Haonan Zhang and Shuai Wang contributed equally to this work.

Email: wuwt@upc.edu.cn, wumb@upc.edu.cn

Supplementary Information Text

The detection of HCHO. Formaldehyde (HCHO) concentrations were measured using a colorimetric method with a UV-Vis spectrophotometer (UV-2700, Shimadzu, Japan), as it could not be detected by ^{1}H NMR spectroscopy. The reagent solution was prepared by dissolving 15 g of ammonium acetate, 0.3 mL of acetic acid, and 0.2 mL of pentane-2,4-dione in 100 mL of water. For the analysis, 0.5 mL of the liquid sample was combined with 2.0 mL of water and 0.5 mL of the prepared reagent solution. The resulting mixture was incubated in a water bath at 35 °C and monitored by UV-Vis absorption spectroscopy until the absorbance at 413 nm reached a stable value.

•OH capture experiment. Coumarin was utilized as the probe for the detection of •OH via the production of 7-hydroxycoumarin (7-HC). Typically, 5 mg catalyst was dispersed in 20 mL coumarin solution (1 mM) and stirred for 1 min. The H_2O_2 (200 μL) was added, and stirred for 30 min at 80 ℃, PL spectra were then measured on the spectrofluorometer (RF-6000, Shimadzu, Japan) after filtering. The excitation wavelength was 330 nm, and the peak value at 454 nm was observed to semi-quantify the concentration of •OH.

•O_2^- capture experiment. •O_2^- was detected by degradation of Nitrotetrazolium Blue chloride (NBT) using NBT as a probe. Generally, 5 mg catalyst was dispersed in 20 mL NBT solution (0.02 mM), stirred for 1 min, and H_2O_2 (200 μL) was added. The total reaction time was 15 min at 80 ℃. After filtering a certain amount every 3 min, it was measured on the UV-vis spectrophotometer (RF-6000, Shimadzu, Japan). The excitation wavelength was 330 nm, the peak value was observed at 260 nm, and the •O_2^- concentration was semi-quantified.

Experimental details of quantification •OH and •O_2^-. Benzoic acid (BA) was used as a probe molecule to react with •OH in catalysts-H_2O_2 suspension under an anaerobic environment. Briefly, 10 mg catalyst was added into a round-bottomed flask with a rubber stopper and followed with Ar gas bubbling to remove oxygen. Subsequently, 20 mL of BA solution (10.4 mmol/L) was continuously bubbled with Ar gas for 30 min to ensure an oxygen-free environment and then pipetted into the round-bottomed flask

with a rubber stopper by an injection syringe. Then the certain concentration of H_2O_2 was pipetted into pollutant suspension to trigger the reaction under shaking throughout the experiment. The reaction solution was collected at a predetermined time, and filtrated through 0.22 μm membranes. HPLC was used to determine the p-hydroxybenzoic acid (p-HBA) concentration. The mobile phase was acetonitrile: water (30 : 70, $v_1 : v_2$) and the detection wavelength was 270 nm. Injection volume, flow rate of mobile phase, and column temperature were fixed at 10 μL, 1.0 mL/min and 35 °C, respectively. Given that 5.87 ± 0.18 moles •OH reacting with BA produced one mole p-HBA. The cumulative •OH concentration is therefore approximately 5.87 times that of p-HBA concentration. Given the fact that 1 mol Nitroblue tetrazolium (NBT) can react with 4 mol $•O_2^-$. We quantified the concentration of generated $•O_2^-$ in the Fenon suspension by recording the residual concentration of NBT on a UV-vis spectrophotometer (maximum absorbance at 260 nm). The experimental procedure was similar as •OH determination experiments except replacing BA with NBT.

The selectivity of oxidative activation H_2O_2 (to $•O_2^-$) and reductive activation H_2O_2 (to •OH) within 90 minutes, were calculated by the following eq. S1 and eq. S2, respectively.

$$\text{Selectivity (to } •O_2^-\text{) (\%)} = 100 * [•O_2^-] / ([•O_2^-] + [•OH]) \qquad \text{eq. S1}$$

$$\text{Selectivity (to •OH) (\%)} = 100 * [•OH] / ([•O_2^-] + [•OH]) \qquad \text{eq. S2}$$

$[•O_2^-]t$ and $[•OH]t$ referred to the concentrations of generated $•O_2^-$ and •OH in 90 minutes, respectively.

The detection of residual H_2O_2. After the reaction, the solution was diluted 10 times with water, then 0.1 mol/L H_2SO_4 and 0.05 mol/L potassium titanium oxalate were added, stirred for one minute, and the absorbance was measured by UV.

H_2O_2 utilization. The H_2O_2 utilization rate is calculated by the following formula:

$$H_2O_2 \text{ utilization} = (1n_{CH3OH} + 2n_{HCHO} + 3n_{HCOOH} + 4n_{CO2}) / n_{consumed\ H2O2}$$

Temperature programmed desorption (TPD). Temperature programmed desorption (TPD) was measured on Micromeritics AutoChemHP-2950. The sample was pretreated in He flows under 500 ℃ for 40 min; and 10% CH_4/He (10% NH_3/He, 10% H_2/He) was used for absorption of the CH_4 (NH_3, H_2) on the sample with 10 mL/min for 1 h,

followed by 1 h He flow with a flow rate of 50 mL/min at 80 °C for removing the physically adsorbed oxygen molecules. CH_4-TPD (NH_3-TPD, H_2-TPR) was measured in He flow at a rate of 50 mL/min. The initial temperature is 80 °C, and the heating rate of 10 °C/min increases to 300 °C. Desorbed CH_4 (NH_3, H_2) was monitored by a thermal conductivity detector (TCD).

Density functional theory (DFT) calculations. All computations using spin polarised density functional theory (DFT) were carried out with the Vienna ab initio simulation package (VASP 5.4.4). The Perdew-Burke-Ernzerhof (PBE) functional based on generalized gradient approximation (GGA) was utilized to describe the exchange-correlation energy. The projector augmented wave (PAW) pseudopotential was implemented to account for the interaction between ions and electrons. The Grimme approach (DFT-D3) was employed to correct for weak van der Waals interactions. A Gamma k-point of 1 × 1 × 1 was used to sample the Brillouin zone. The kinetic energy cutoff was set to 400 eV, and atomic coordinates were relaxed until the maximum force was below 0.05 eV/Å. The electron self-consistent cycle convergence criterion was set to 1×10^{-4} eV.

The adsorption free energy (ΔG) is calculated by $\Delta G = \Delta E + \Delta ZPE - T\Delta S$, where ΔE is the DFT based adsorption energy, ΔZPE and TΔS are the correction of zero point energy and entropy, respectively.

The MFI-type zeolite unit cell including 288 atoms was chosen as the computational model of ZSM-5 with a lattice constant of 20.09 Å × 19.74 Å × 13.14 Å from the Database of Zeolite Structures. Two Si atoms located at the T8 and T12 sites within two adjacent 5-membered rings of the straight channel were replaced by two Al atoms and the Brønsted acid sites of ZSM-5 were created by adding two H atoms bound to bridge O atoms of two -Si-O-Al- units to compensate the framework negative charge with Al substitution.

Supplementary Information Figures

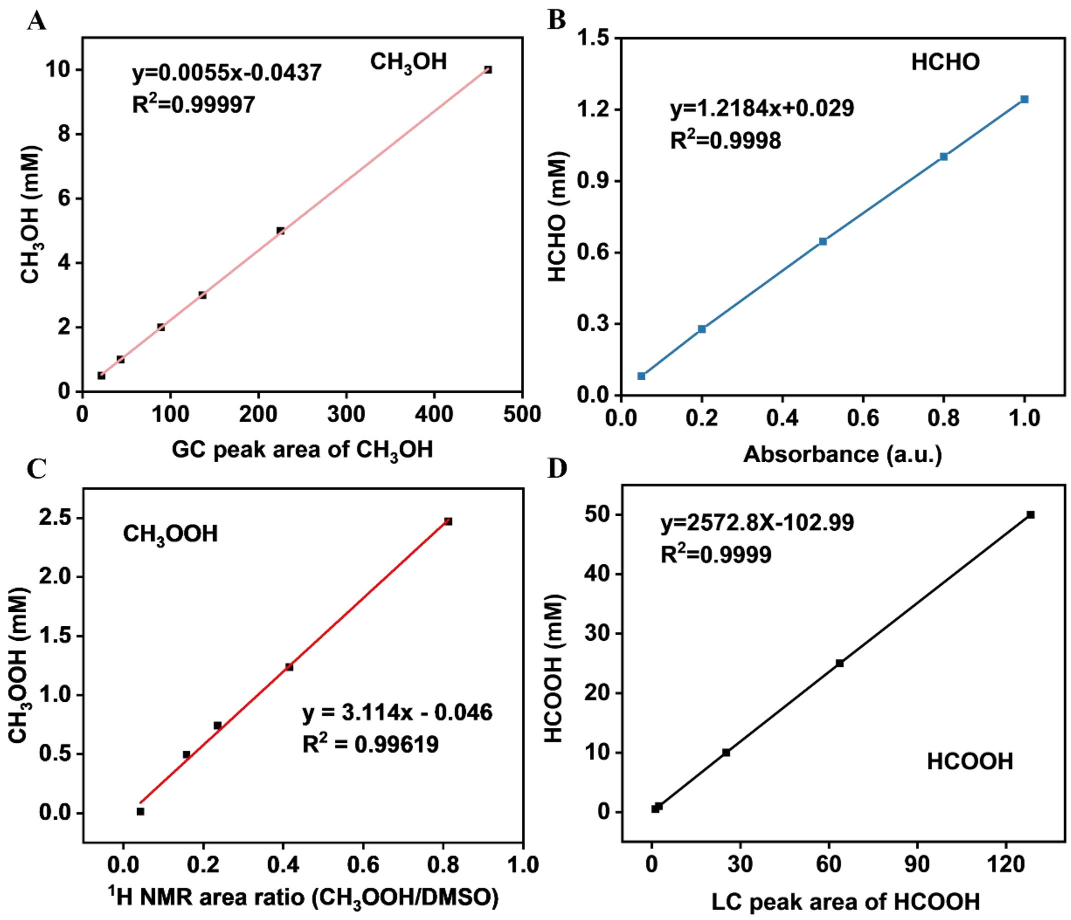

Supplementary Figure 1 | Standard curve. (A) Standard curve of CH_3OH in gas chromatography. (B) Standard curve of HCHO in UV-vis spectra. (C) Standard curve of CH_3OOH in ^{1}H-NMR. (D) Standard curve of formic acid in liquid chromatography.

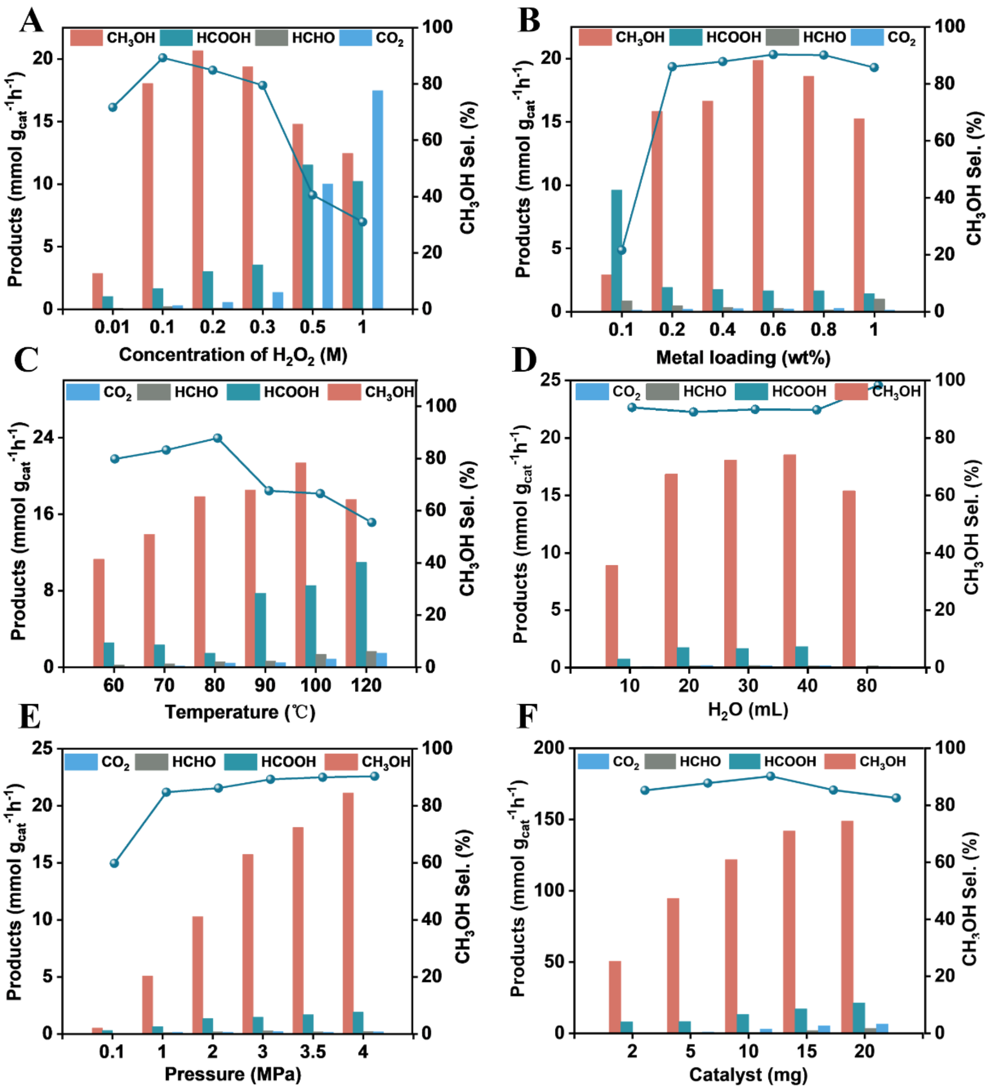

Supplementary Figure 2 | Standard curve and selective oxidation of CH_4 at low temperature under different reaction conditions. (A) Catalytic performance of different concentration of H_2O_2. Reaction conditions: 3.5 MPa CH_4, 20 mL H_2O, 10 mg catalyst, 3 h, 80 °C. (B) Catalytic performance of different mental loading amounts. Reaction conditions: 3.5 MPa CH_4, 20 mL 0.1 M H_2O_2, 10 mg catalyst, 3 h, 80 °C. (C) Catalytic performance of different temperatures. Reaction conditions: 3.5 MPa CH_4, 20 mL 0.1 M H_2O_2, 10 mg catalyst, 3 h. (D) Catalytic performance of different H_2O volume. Reaction conditions: 3.5 MPa CH_4, 10 - 80 mL 0.1 M H_2O_2, 10 mg catalyst, 3 h, 80 °C. (E) Catalytic performance of different reaction pressure. Reaction conditions: 0.1 - 4.0 MPa CH_4, 20 mL 0.1 M H_2O_2, 10 mg catalyst, 3 h, 80 °C. (F) Catalytic performance of different quality of catalysts. Reaction conditions: 3.5 MPa CH_4, 20 mL 0.1 M H_2O_2, 2-20 mg catalyst, 3 h, 80 °C.

Note: The results showed that the liquid products contained C_1 oxygen-containing compounds, such as CH_3OH, HCOOH, and HCHO. Without Cu metal, a large amount of HCOOH appeared in the reaction

product. Increasing the Cu load to 0.2 wt%, the total liquid yield improved and it increased the selectivity of CH_3OH from 20% to 90%, suggesting that the introduction of Cu promote CH_3OH generation selectively. Further increases in Cu loading continued to improve both total yield and CH_3OH selectivity, but not significantly, indicating that Fe single atomic sites are crucial for methane activation while Cu single atomic sites play a key role in improving CH_3OH selectivity. However, excessive Cu loading may compete with Fe for methane adsorption and hinders its activation by Fe, leading to decreased yield. The amount of catalyst also affects the CH_3OH selectivity. The peak selectivity occurs at 10 mg of catalyst. However, using too much catalyst can lead to over-oxidation of CH_3OH due to the excessive decomposition of H_2O_2. Furthermore, the solvent amount used in the reaction influenced both the catalytic yield and selectivity of CH_3OH. As H_2O addition increased from 10 mL to 40 mL, there was a proportional increase in CH_3OH yield. At 80 mL conditions, CH_3OH selectivity approached 100%, albeit with a slight decrease in total yield. This is because the catalyst is more evenly dispersed in H_2O which can carry away the fresh product in time, and can better decompose and utilize H_2O_2, avoiding the peroxide process.

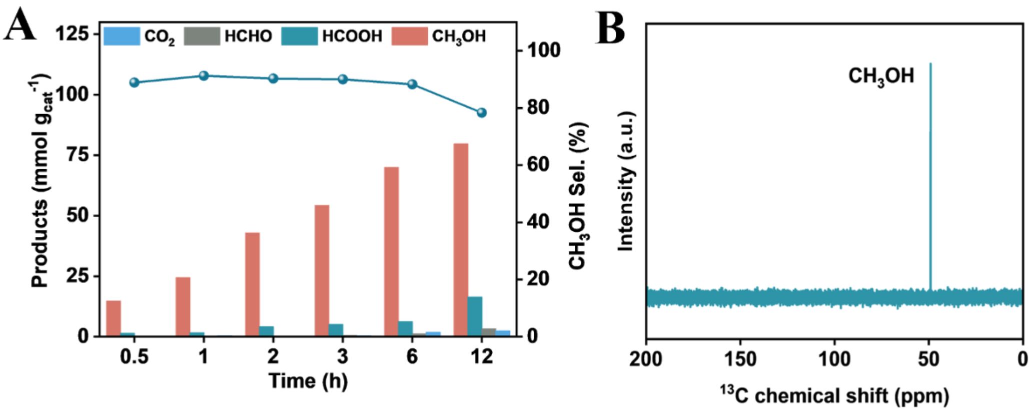

Supplementary Figure 3 | (A) Catalytic performance of different time. Reaction conditions: 3.5 MPa CH_4, 20 mL H_2O, 10 mg catalyst, 80 °C, 0.1 M H_2O_2. (B) ^{13}C NMR spectra of the products.

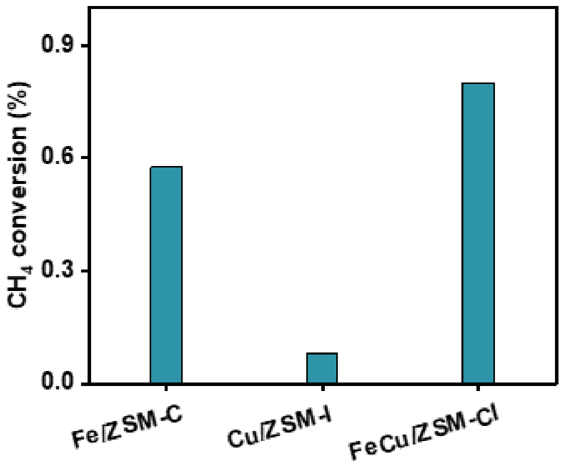

Supplementary Figure 4 | CH_4 conversion of different catalysts. Reaction Condition: 10 mg catalysts dispersed in 20 mL of 0.1 mol/L H_2O_2 aqueous solution, 3 hours, 80 °C and 3.5 MPa CH_4.

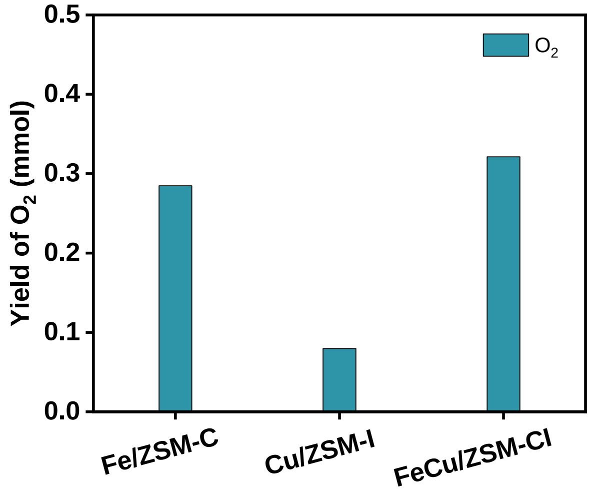

Supplementary Figure 5 | The amount of O_2 produced by the H_2O_2 self-decomposition.

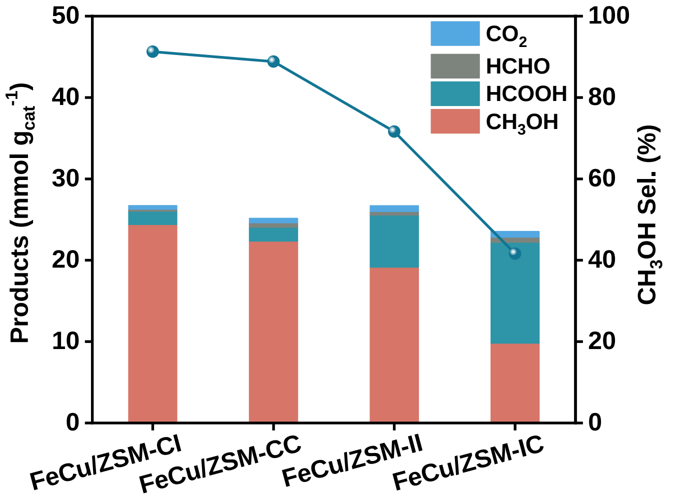

Supplementary Figure 6 |Comparison of CH_3OH selectivity of as-prepared samples under equivalent activity.Reaction conditions: 10 mg of cat., 20 mL of H_2O as solvent, 0.1 M of H_2O_2 as oxidant, 3.5 MPa CH_4, 80 ºC, 1 h for FeCu/ZSM-CI, 2.2 h for FeCu/ZSM-CC, 2.4 h for FeCu/ZSM-II, 2.9 h for FeCu/ZSM-IC.

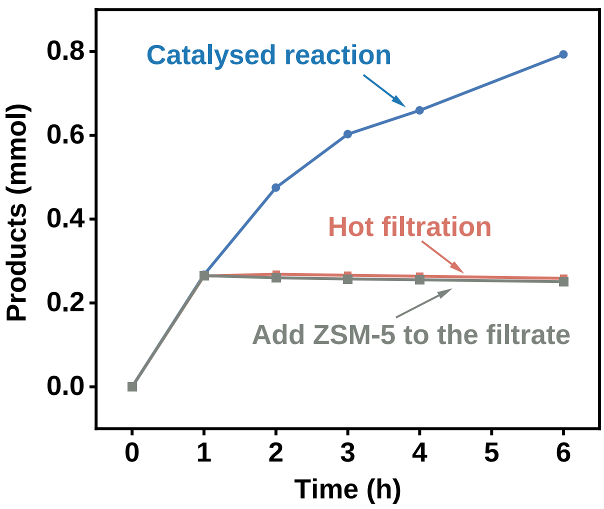

Supplementary Figure 7 | Time-dependent conversion and hot filtration test of the reaction.

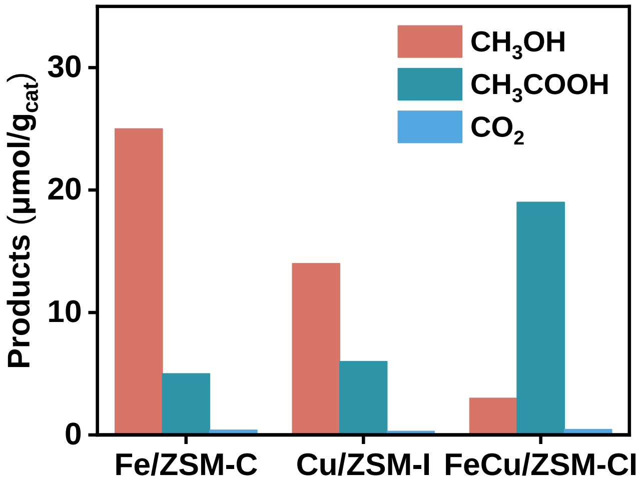

Supplementary Figure 8 |The catalytic oxidation performance of CH_4 when O_2 acts as the oxidant. Reaction conditions: 15 mg catalysts dispersed in 15 mL aqueous solution, 210 °C, 3.5 MPa CH_4, and 0.5 MPa O_2 maintain 24 h.

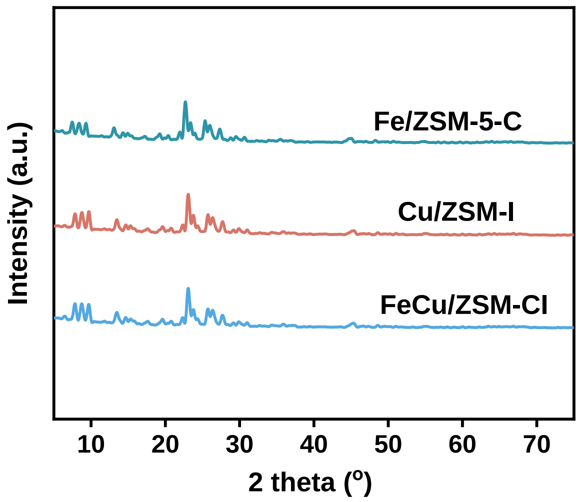

Supplementary Figure 9 | XRD patterns of different catalysts.

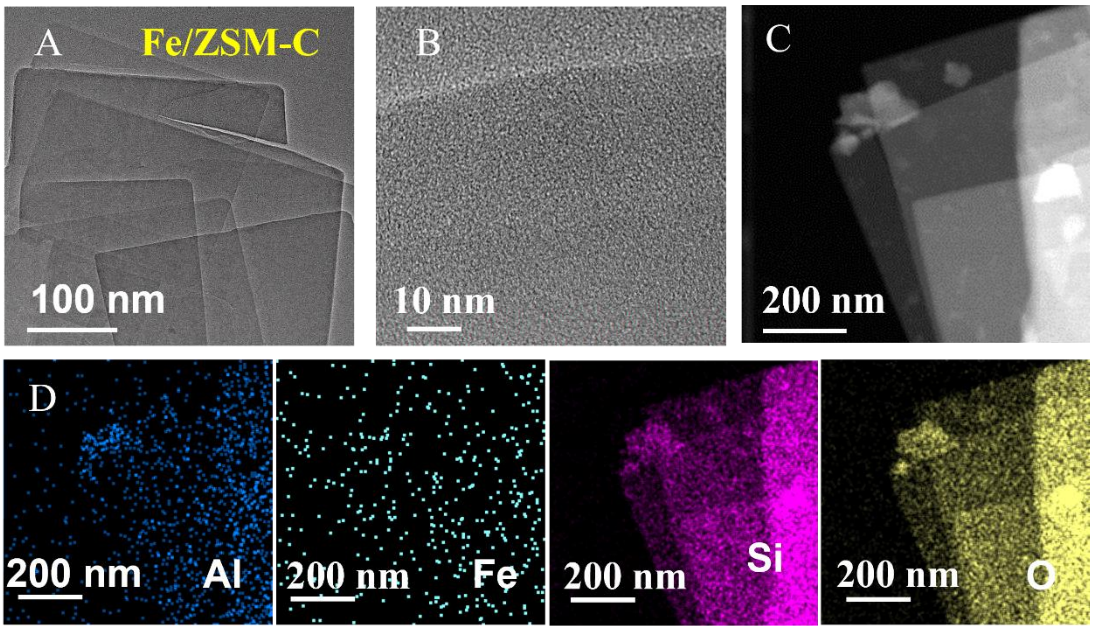

Supplementary Figure 10 | HRTEM and HRTEM-EDS-mapping images of Fe/ZSM-C. Where green, blue, purple and yellow represent Fe, Al, Si and O, respectively.

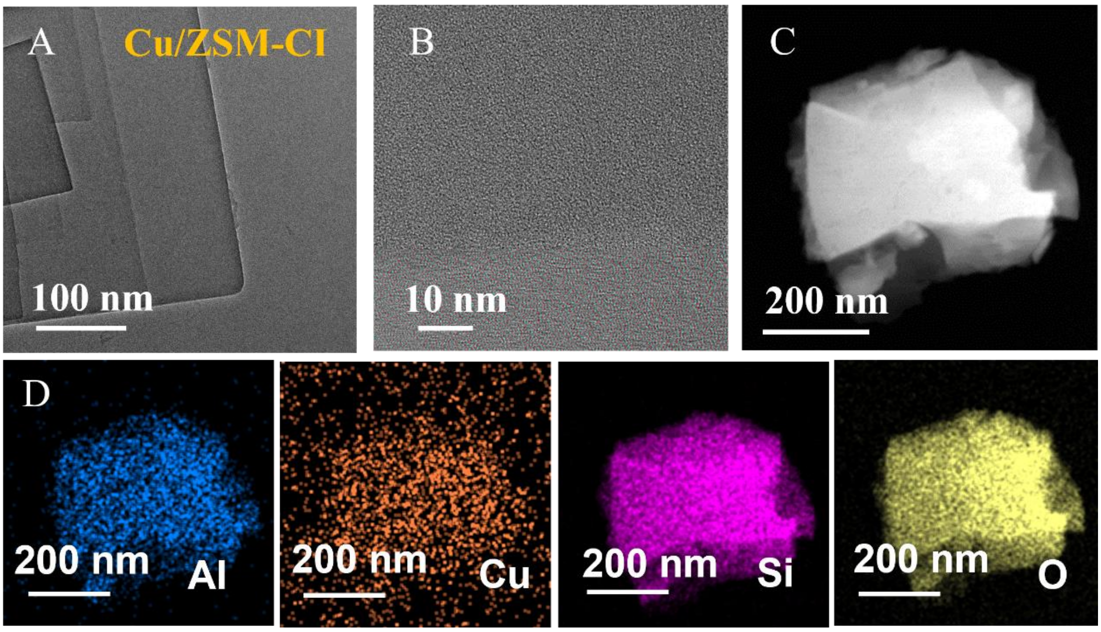

Supplementary Figure 11 | HRTEM and HRTEM-EDS-mapping images of Fe/ZSM-C. Where orange, blue, purple and yellow represent Cu, Al, Si and O, respectively.

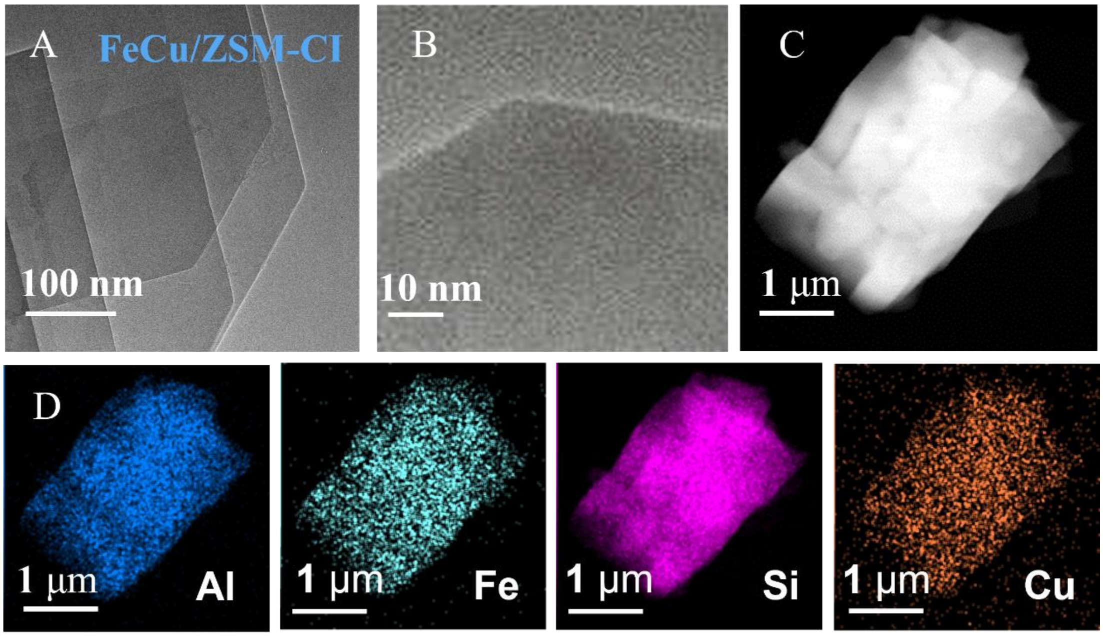

Supplementary Figure 12 | HRTEM and HRTEM-EDS-mapping images of Fe/ZSM-C. Where green, orange, blue, purple and yellow represent Fe, Cu, Al, Si and O, respectively.

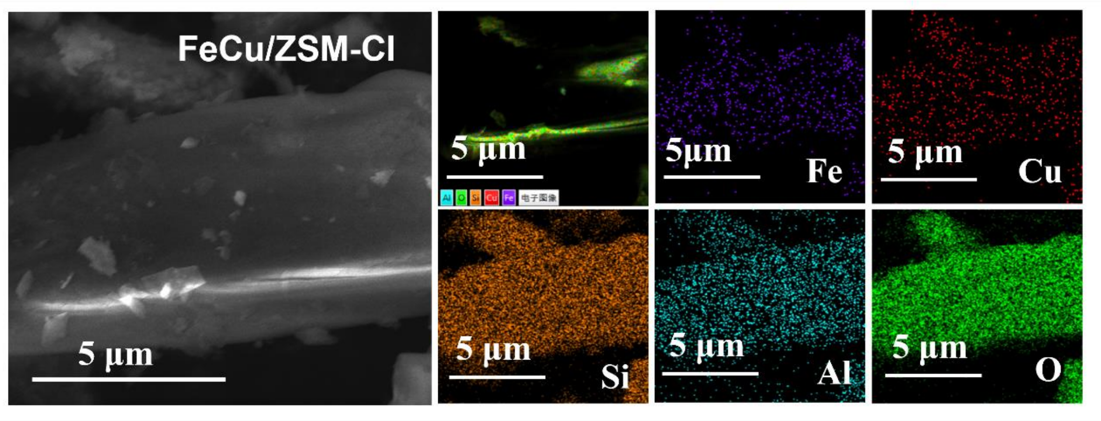

Supplementary Figure 13 | SEM image and SEM-EDS-mapping images of FeCu/ZSM-CI catalyst.

Where purple, red, orange, dark green and green represent Fe, Cu, Si, Al and O, respectively.

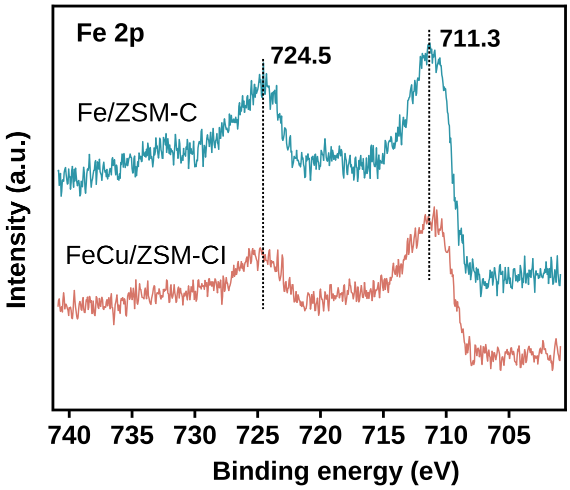

Supplementary Figure 14 | Fe 2*p* XPS spectra of FeCu/ZSM-CI.

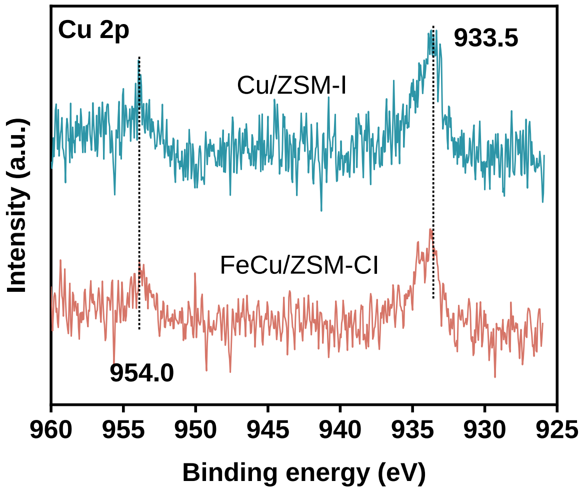

Supplementary Figure 15 | Cu 2*p* XPS spectra of FeCu/ZSM-CI.

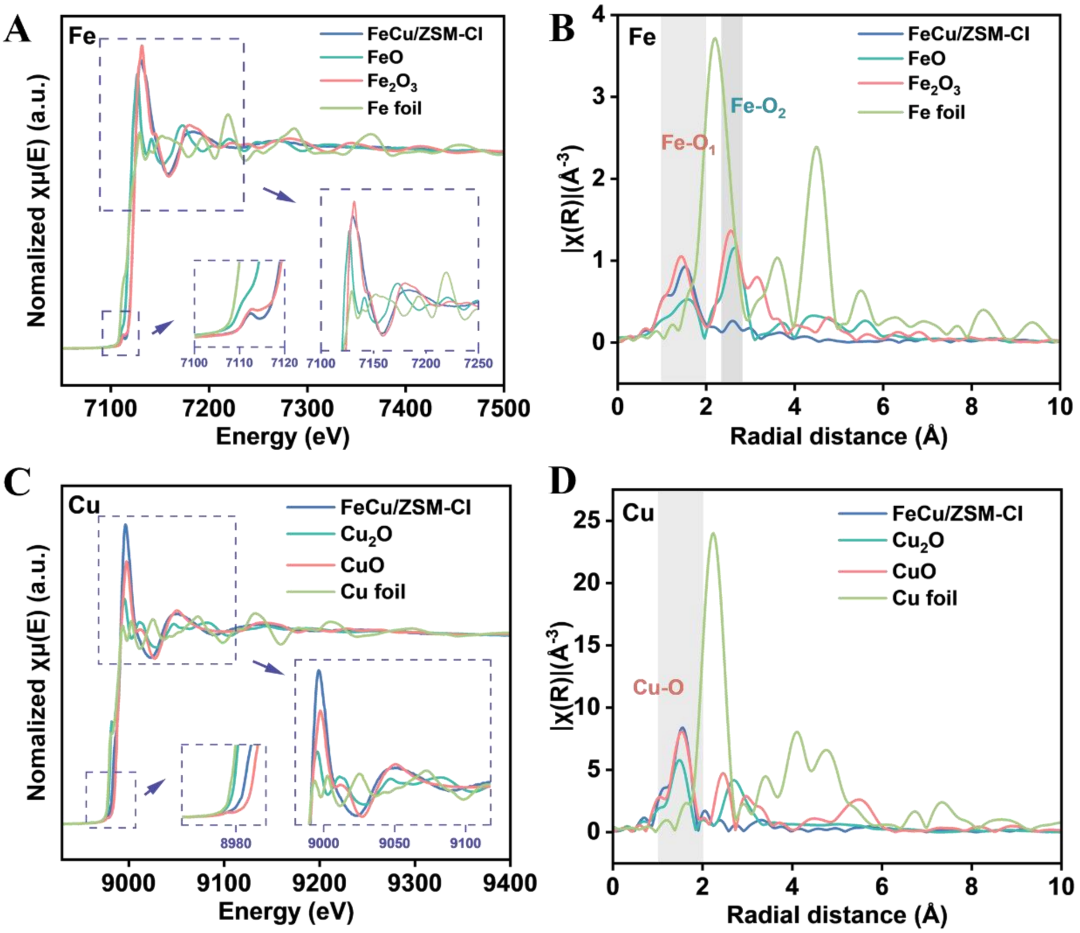

Supplementary Figure 16 | (A) XANES spectra at Fe K-edge of FeCu/ZSM-CI in comparison with Fe foil, FeO and Fe_2O_3. (B) Fourier transform (FT) k^3-weighted EXAFS spectra of FeCu/ZSM-CI in comparison with Fe foil, FeO and Fe_2O_3. (C) XANES spectra at Cu K-edge of FeCu/ZSM-CI in comparison with Cu foil, Cu_2O and CuO. (D) Fourier transform (FT) k^3-weighted EXAFS spectra of FeCu/ZSM-CI in comparison with Cu foil, Cu_2O and CuO.

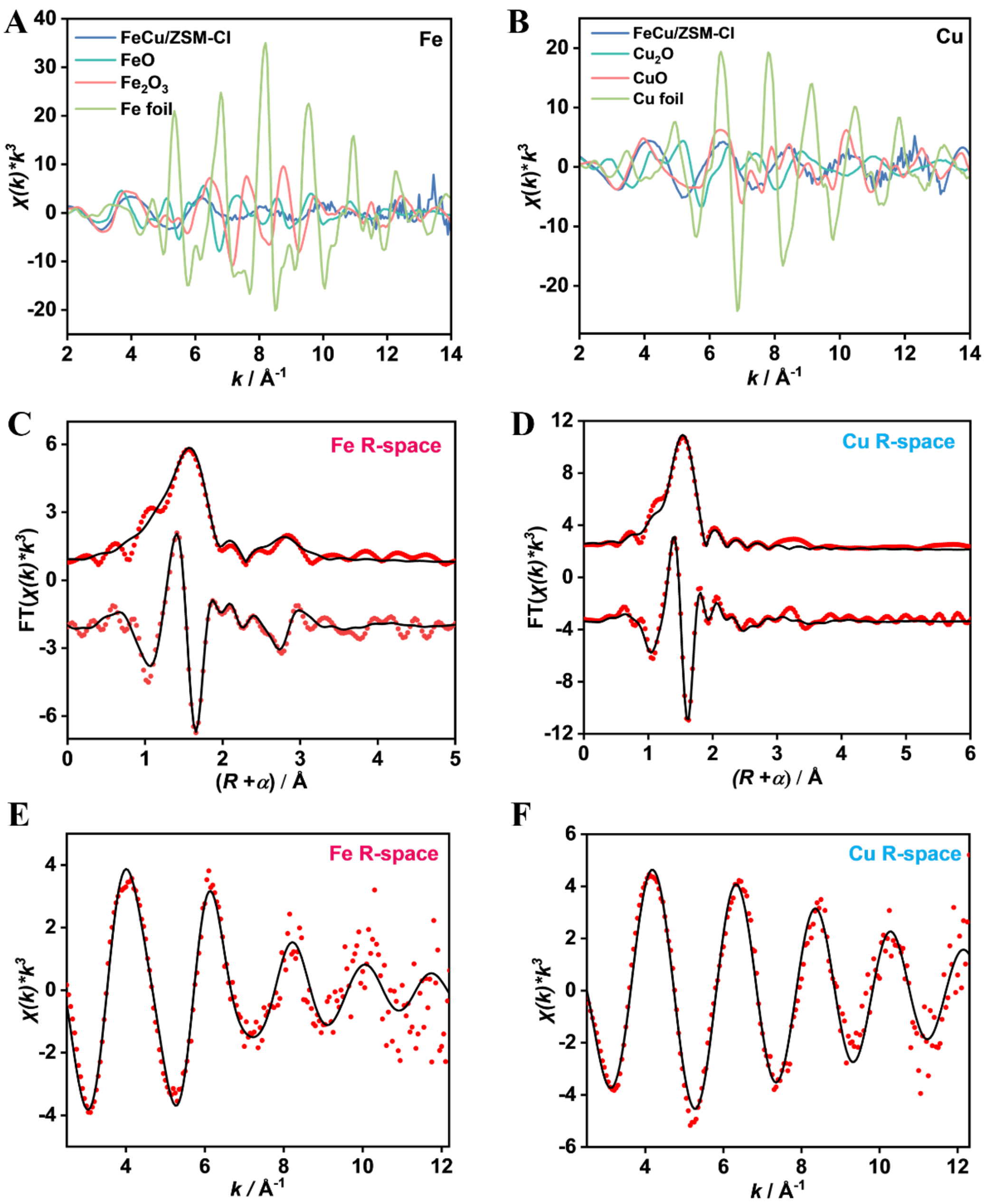

Supplementary Figure 17 | EXAFS fitting analysis of FeCu/ZSM-**CI.** (a-b) Fe/Cu K-edge EXAFS; (c-d) Fe/Cu K-edge EXAFS (points) and curvefit (line) for FeCu/ZSM-CI, shown in k^3 *R*- space (FT magnitude and imaginary component). The dates are k^3-weighted and not phase-corrected. (e-f) Fe/Cu K-edge EXAFS (points) and the curvefit (line) for FeCu/ZSM-CI, shown in k^3-weighted k-space.

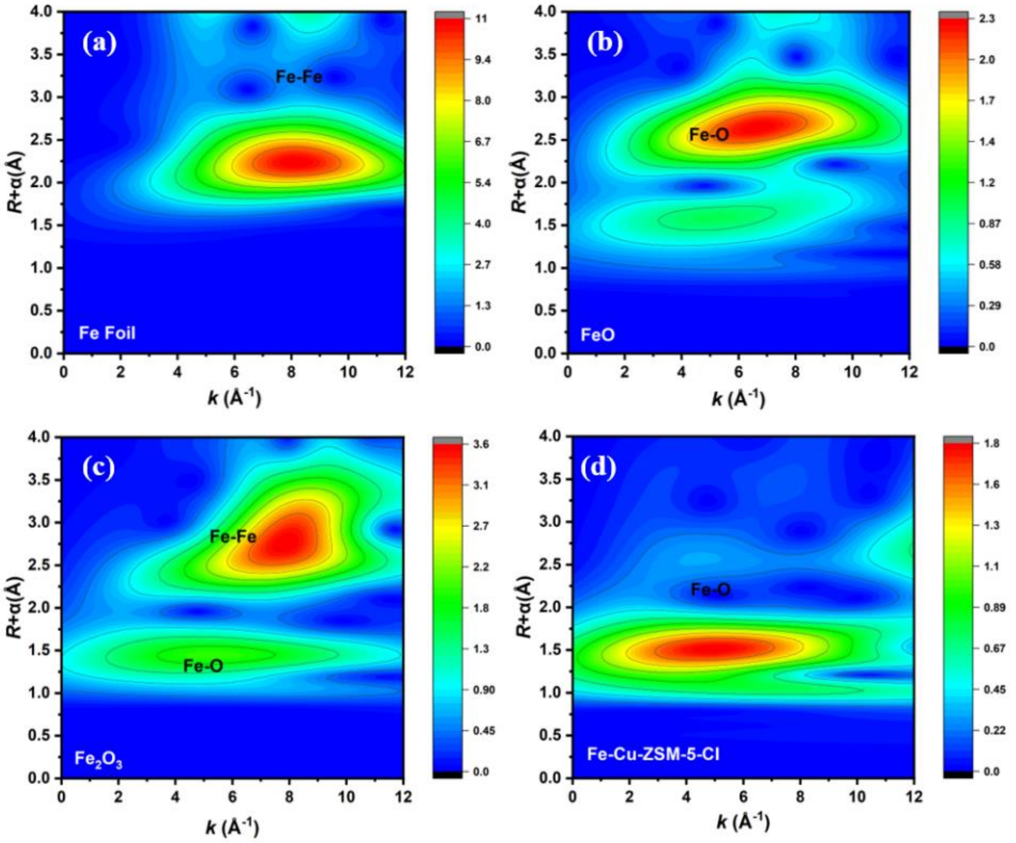

Supplementary Figure 18 | Wavelet transform (WT) analysis of the Fe K-edge EXAFS oscillations of FeCu/ZSM**-CI.** (a) Fe foil, (b) FeO, (c) Fe_2O_3, (d) FeCu/ZSM-CI.

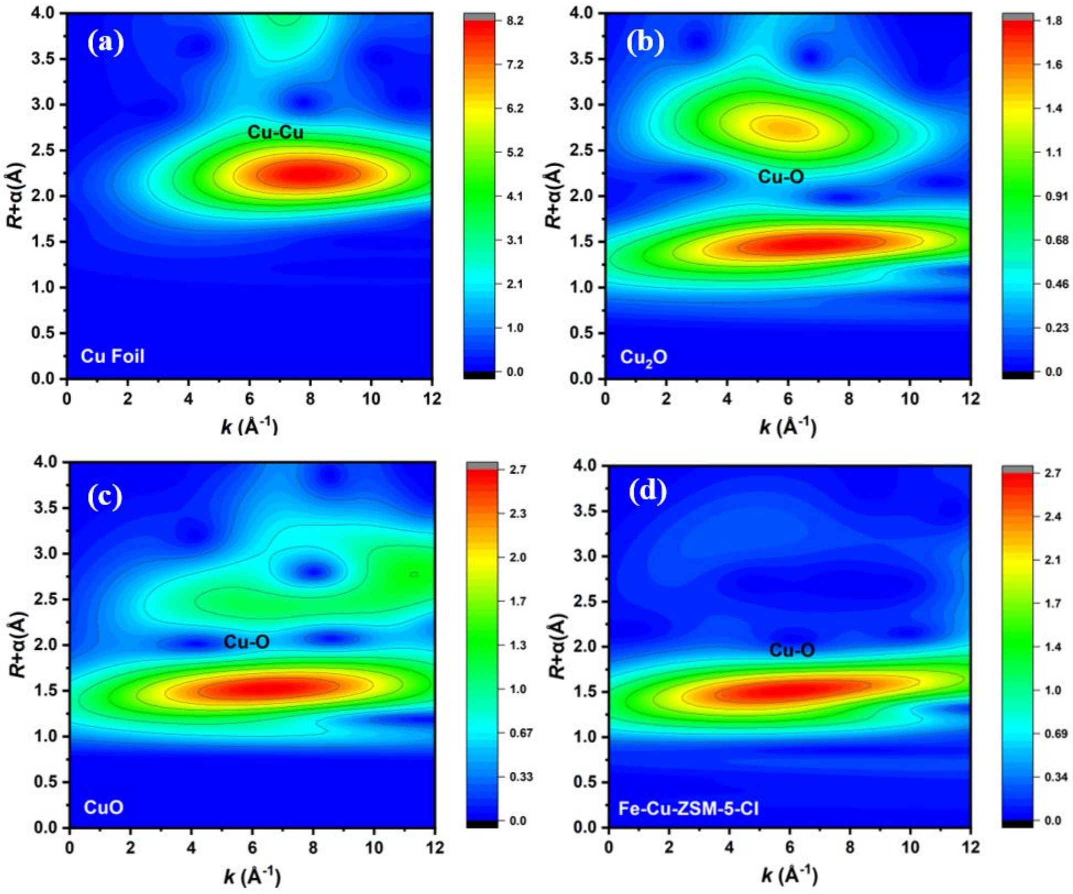

Supplementary Figure 19 | Wavelet transform (WT) analysis of the Cu K-edge EXAFS oscillations of FeCu/ZSM-CI. (a) Cu foil, (b) Cu_2O, (c) CuO, (d) FeCu/ZSM-CI.

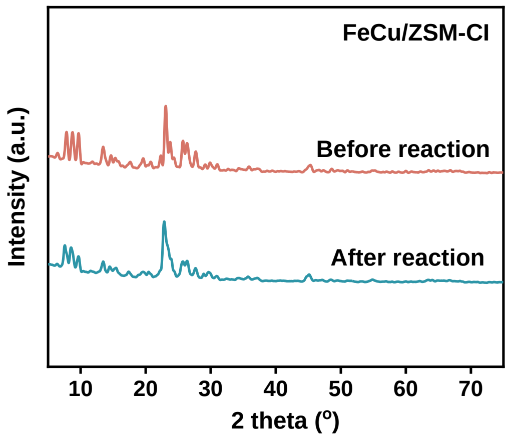

Supplementary Figure 20 | XRD patterns of the fresh catalyst and catalyst after reaction of FeCu/ZSM-CI.

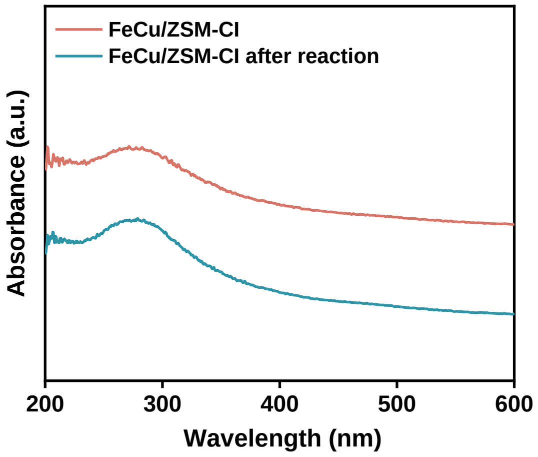

Supplementary Figure 21 | DR UV-vis spectra of the fresh catalyst and catalyst after reaction of FeCu/ZSM-CI.

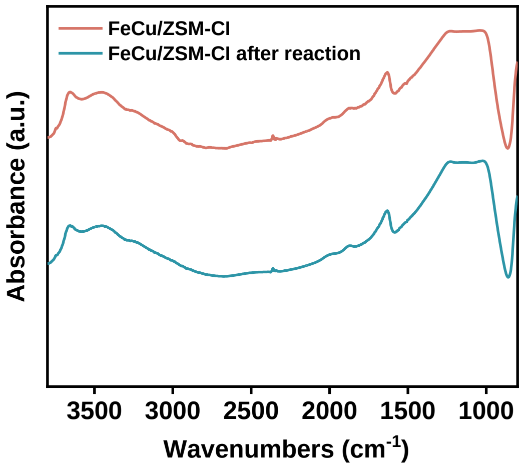

Supplementary Figure 22 | IR spectra of the fresh and catalyst after reaction of FeCu/ZSM-CI.

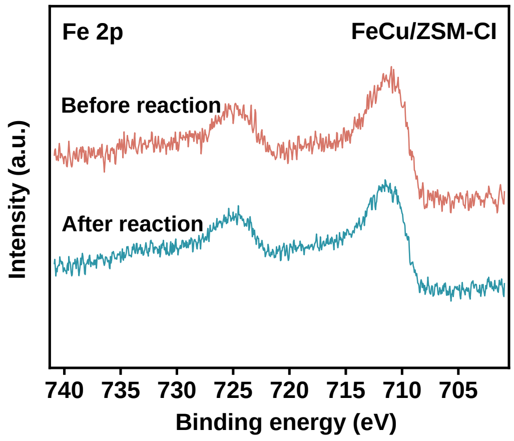

Supplementary Figure 23 | Fe 2p XPS spectra of the fresh and catalyst after reaction of FeCu/ZSM-CI.

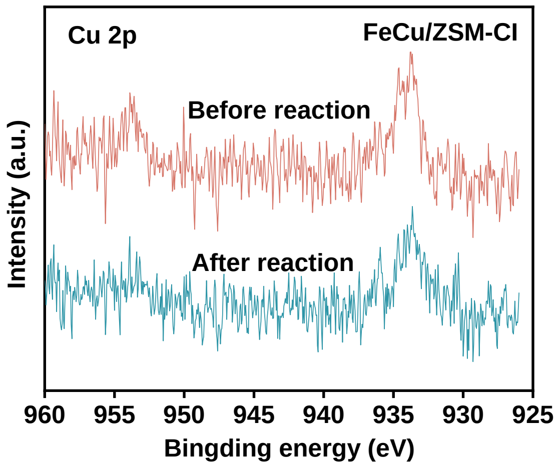

Supplementary Figure 24 | Cu 2p XPS spectra of the fresh and catalyst after reaction of FeCu/ZSM-CI.

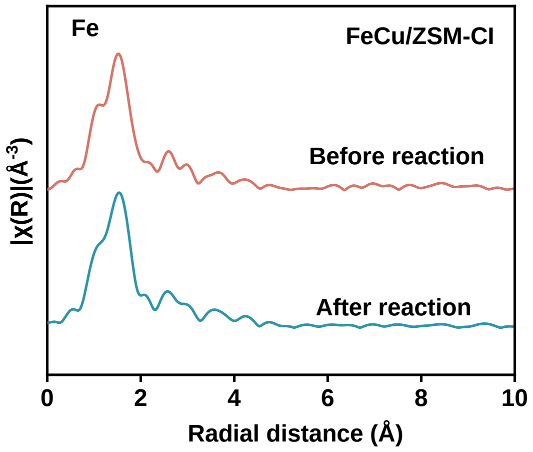

Supplementary Figure 25 | R-space of Fe R-edge EXAFS for the fresh catalyst and catalyst after reaction of FeCu/ZSM-CI.

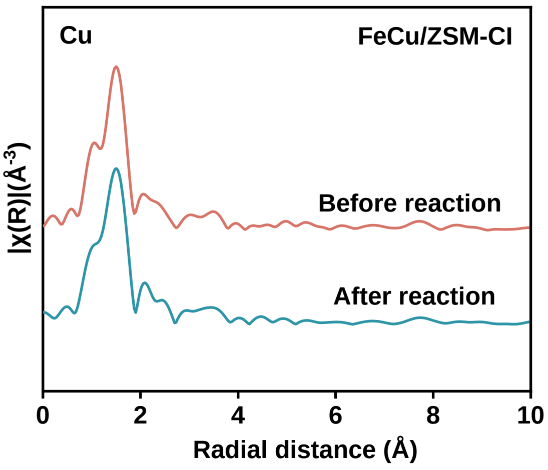

Supplementary Figure 26 | R-space of Cu R-edge EXAFS for the fresh catalyst and catalyst after reaction of FeCu/ZSM-CI.

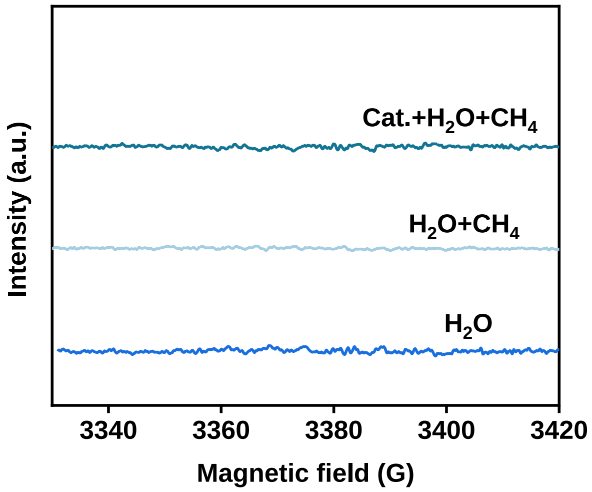

Supplementary Figure 27 | EPR spectra of FeCu/ZSM-CI under different reaction conditions.

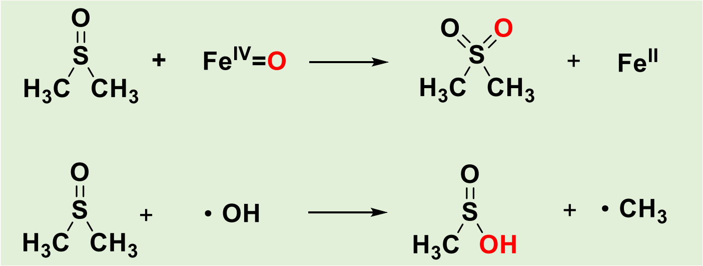

Supplementary Figure 28 | Determination of $Fe^{IV}=O$ by DMSO.

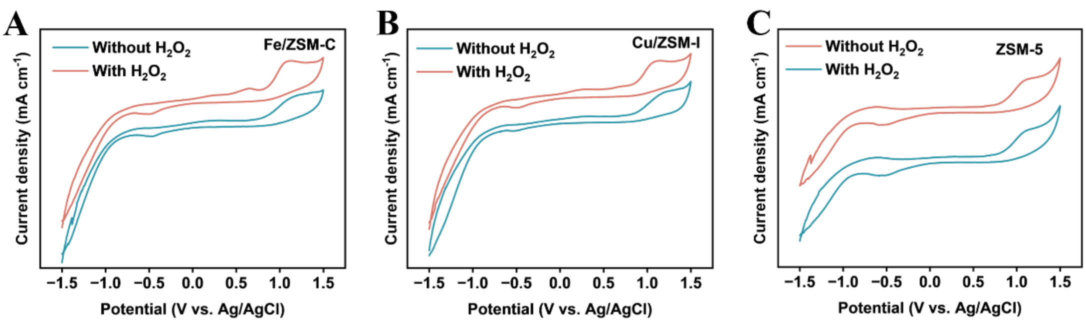

Supplementary Figure 29 | *In-situ* CV tests of catalysts for H_2O_2 activation.

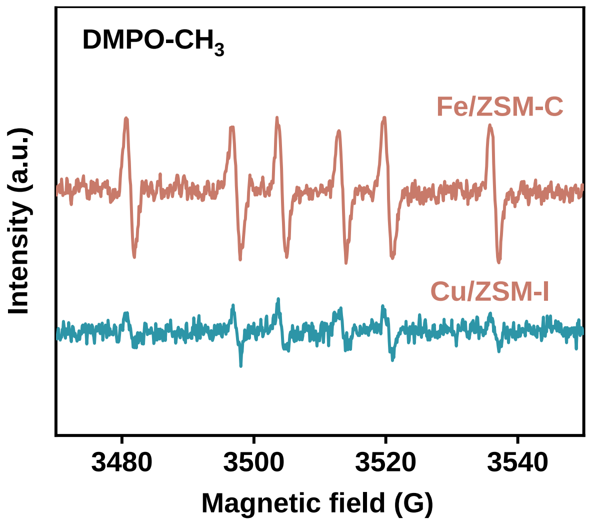

Supplementary Figure 30 | EPR spectra of •CH_3 under Fe/ZSM-C and Cu/ZSM-I.

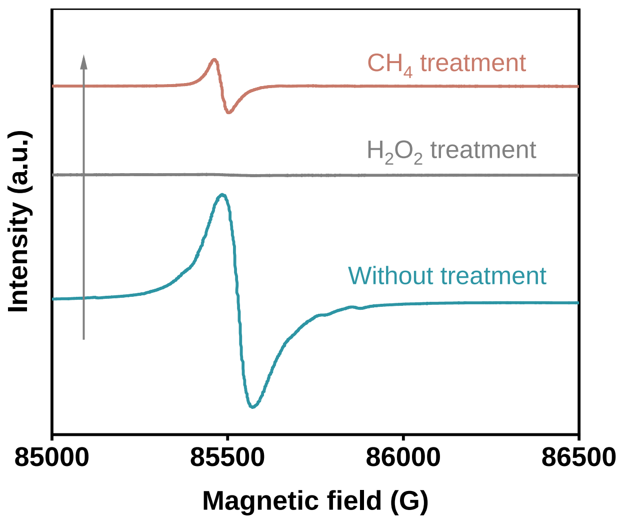

Supplementary Figure 31 | *In-situ* High-field EPR of the catalyst during reaction. Reaction conditions: 463 K, 2 h, test conditions: 15 K, microwave frequency: 240 Hz, Fe/ZSM-C.

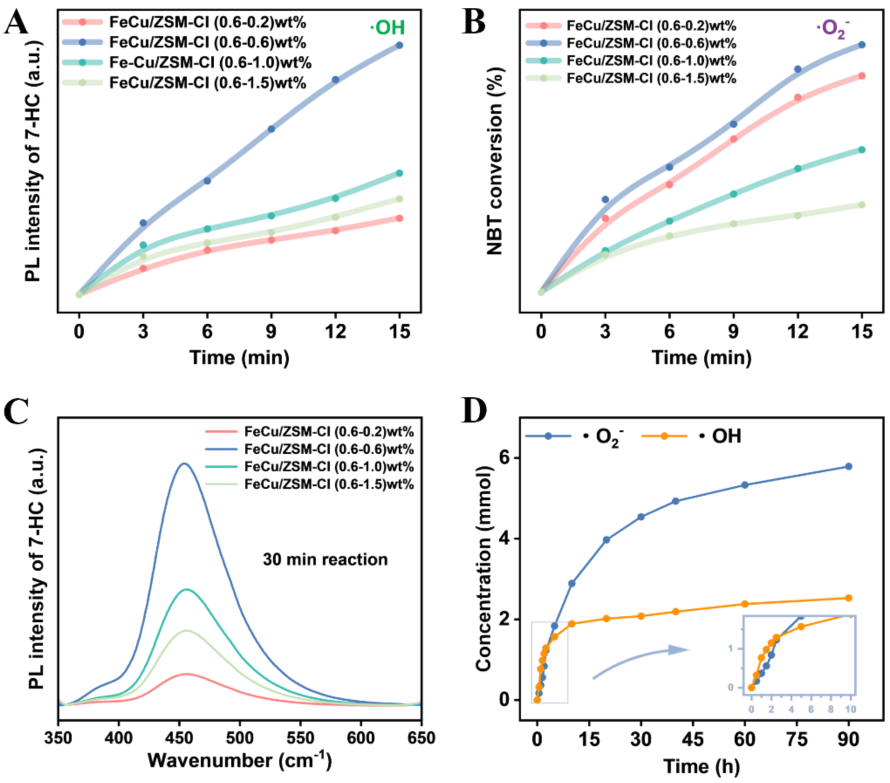

Supplementary Figure 32 | Experiment on H_2O_2 decomposition. (a) $\bullet O_2^-$ under catalysts of different metal contents with time. (b) •OH capture under different catalysts of metal contents with time. (c) PL spectra of 7-hydroxycoumarin by •OH under different catalysts of metal contents. (d) The ratio of H_2O_2 to free radicals.

Note: •OH were initially detected at a faster rate and generated more rapidly than •OOH. This phenomenon can be attributed to the fact that •OH is predominantly produced through the decomposition of H_2O_2 at the external large pores' copper sites, whereas •OOH primarily arises from H_2O_2 decomposition at the inner small pores' iron sites. The diffusion characteristics of H_2O_2 vary between small and large pore sizes, leading to differential reaction rates across various active sites; consequently, •OH is preferentially formed and diffuses into the solution without being consumed, while •OOH formation occurs relatively slowly. After a reaction duration of 15 min, it became evident that the generation rate of •OOH surpassed that of •OH, primarily due to enhanced H_2O_2 decomposition at the iron site. By strategically adjusting the positions of different metal sites on ZSM-5 zeolite, one can effectively control both the generation pathways and quantities of free radicals.

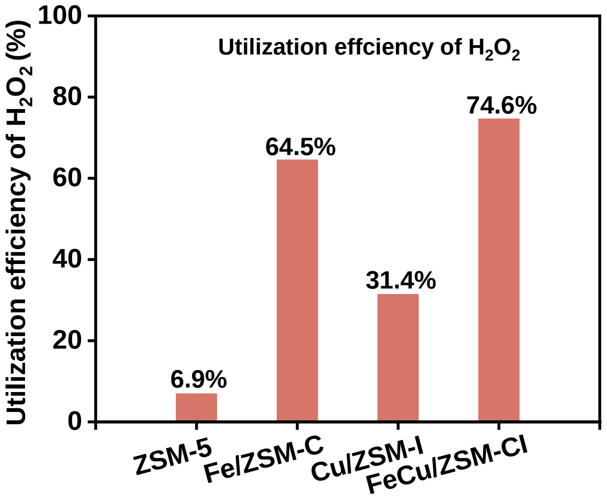

Supplementary Figure 33 | Utilization efficiency of H_2O_2.

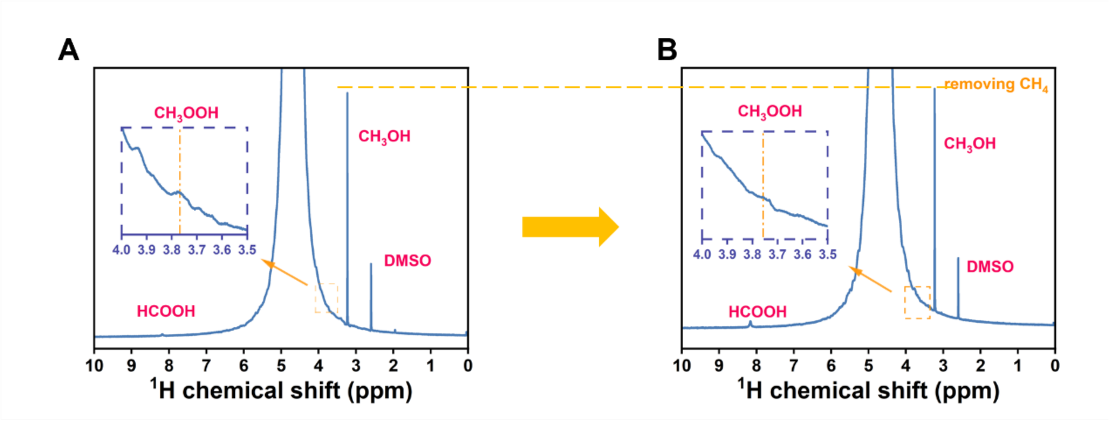

Supplementary Figure 34 | ^{1}H NMR of oxidation products under Standard reaction condition. (a) ^{1}H NMR spectra after reaction. (b) ^{1}H NMR spectrum of the solution after reaction, with the removal of methane gas and subsequent reaction. Reaction conditions: In a 50 mL high-pressure reactor, 20 mL H_2O_2 solution (concentration is 0.1 mol/L), reaction temperature is 80 °C, catalyst dosage is 10 mg, CH_4 is 3.5 MPa, reaction time is 3 h.

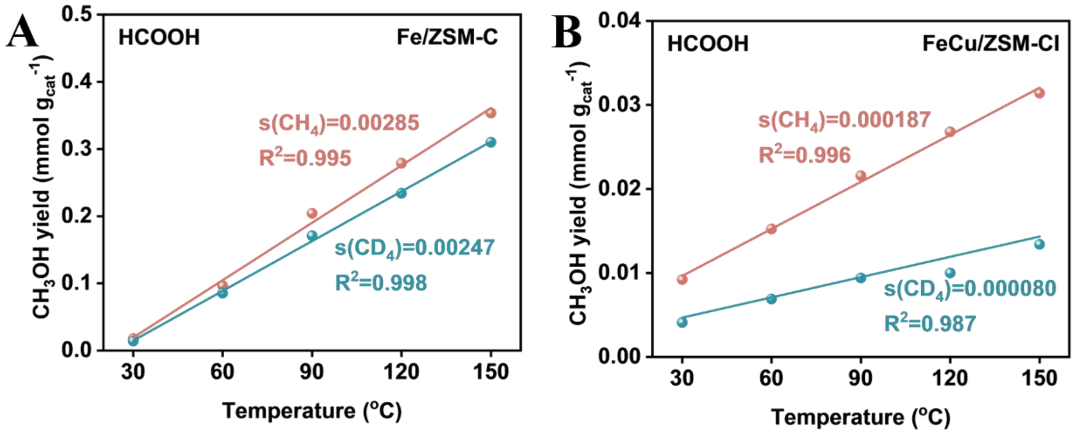

Supplementary Figure 35 | Kinetic isotope effect experiment of HCOOH over Fe/ZSM-C (A) and FeCu/ZSM-CI (B).

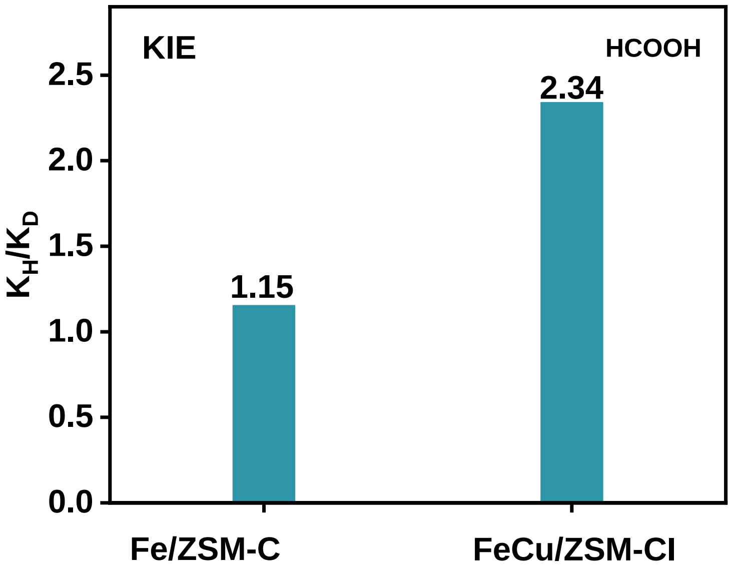

Supplementary Figure 36 | Kinetic isotope effect experiment of HCOOH production over Fe/ZSM-C and FeCu/ZSM-CI.

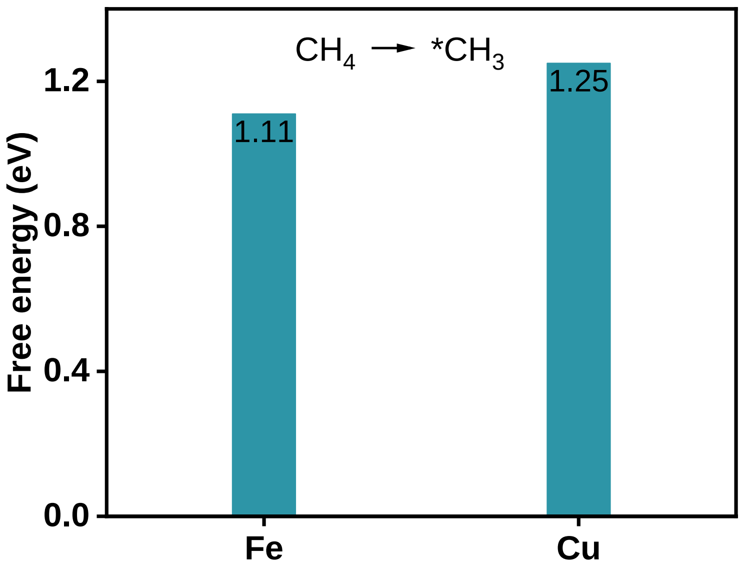

Supplementary Figure 37 | Comparison of free energy barriers for CH_4.

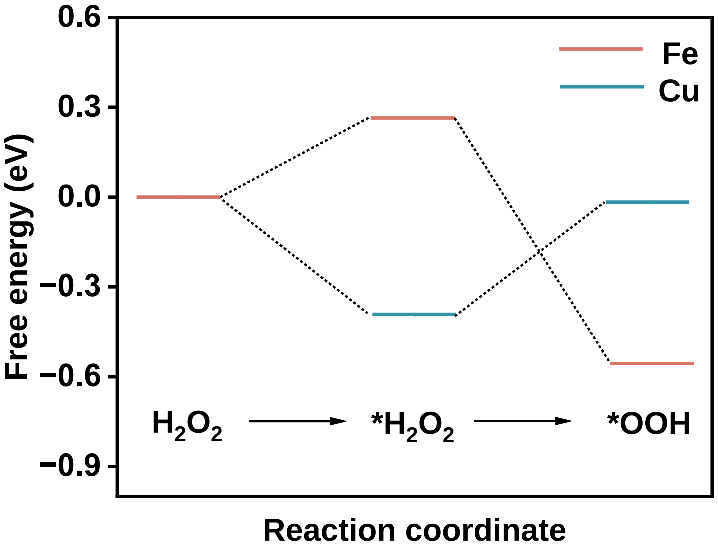

Supplementary Figure 38 | The change of free energy of H_2O_2 to *OOH.

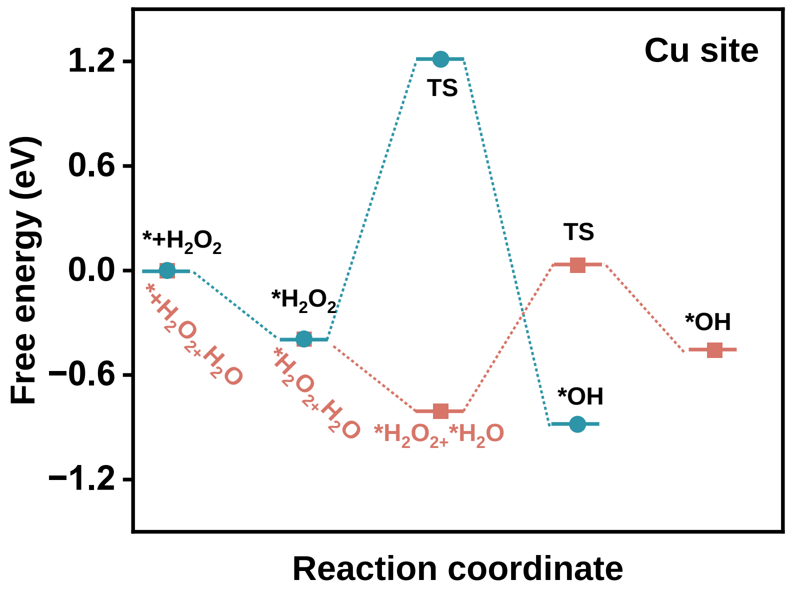

**Supplementary Figure 39 | ** The influence of H_2O on the free energy of decomposition of H_2O_2 into *OH.

Supplementary Information Tables

Supplementary Table S1 | Comparative experiment results obtained by replacing reactants.

Reaction condition	Productivity (mmol g_{cat}^{-1} h^{-1})				Selectivity of CH_3OH (%)	Gross productivity /mmol g_{cat}^{-1} h^{-1}
	CH_3OH	HCHO	HCOOH	CO_2		
Without H_2O_2	No product				--	--
Without CH_4	No product				--	--
Without catalyst	No product				--	--
Without H_2O_2, 0.5 MPa O_2	No product				--	--
Additional 0.5 MPa O_2	21.45	0.11	2.04	0.15	90.3	23.76

Note: Reaction conditions: 3.5 MPa CH_4, 20 mL 0.1 M H_2O_2, 10 mg catalyst, 3 h, 80 °C.

Table S2. Comparison of methane conversion rates.

Catalyst	Conversion of CH_4 (%)	Reference
FeCu/ZSM-CI	1.19	This work
Pd_1-ZSM-5[(1)]	0.02	Angew. Chem. Int. Ed. 2024,64, e202315343
PdCu/Z-5[(2)]	0.17	Angew. Chem. Int. Ed. 2022, 61, e202204116
Au/MOR[(3)]	0.25	J. Am. Chem. Soc. 2023, 145, 12928−12934
CeO_2@PdO@FeOx[(4)]	0.02	J. Am. Chem. Soc. 2024, 146, 25870−25877
$BiVO_4$@Au[(5)]	0.018	Angew. Chem. Int. Ed. 2024, 64, e202419282
Ru/ZnO[(6)]	0.013	Nat. Sustain. 2024,7,1171-1181
MoS_2[(7)]	0.067	Nat Catal. 2023, 6, 1052-1061
Au-ZSM-5[(8)]	0.22	Nature Catal. 2022, 5, 45-54
Zn-O-Fe[(9)]	0.01	Angew. Chem. Int. Ed. 2025, 64, e202510241
Cu_9S_6-Cu-C_3N_4[(10)]	0.024	Nature Commun. 2024, 15, 10451

Supplementary Table S3 | Comparisons with the representative catalytic performances on the yield and selectivity of CH_3OH.

Entry	Catalyst	Reaction temperature (°C)	Concentration of H_2O_2 (mol/L)	Reaction condition	CH_3OH selectivity in all oxygenates (%)	CH_3OH space-time yield (mmol g_{cat}^{-1} h^{-1})	H_2O_2 utilization (%)	Reference
1	FeCu/ZSM-CI	80	0.1	10 mg catalyst, 3.5 MPa CH_4, 20 mL H_2O, 3 h	90.1	20.2	74.6	This work
2	Ag_1-Cu_1/ZSM-5 hetero-SAC	70	0.489	22 mg catalyst, 3 MPa CH_4, 21.05 mL H_2O, 0.5 h	81	40.2	7.32	(11)
3	Cu-Fe (2/0.1)/ZSM-5	50	0.5	10 mg catalyst, 3 MPa CH_4, 20 mL H_2O, 1 h	80	3.9	0.68	(12)
4	Cu_1/ZSM-5	50	0.5	28 mg catalyst, 3 MPa CH_4, 10 mL H_2O, 0.5 h	74	8.2	4.72	(13)
5	CuFe/ZSM-5	50	0.5	27 mg catalyst, 3.05 MPa CH_4, 10 mL H_2O, 0.5 h	85	8.2	3.39	(14)
6	Fe/ZSM-5	50	0.5	27 mg catalyst, 3.05 MPa CH_4, 10 mL H_2O, 0.5 h	12	1.2	7.45	(14)

7	Au-Pd/TiO_2	50	0.5	10 mg catalyst, 3 MPa CH_4, 10 mL H_2O, 0.5 h	12	0.14	0.32	(15)
8	Pd_1/ZSM-5	50	0.5	28 mg catalyst, 3 MPa CH_4, 20 mL H_2O, 0.5 h	86.4	0.55	0.11	(16)
9	Rh_1/ZSM-5	70	0.5	30 mg catalyst, 3 MPa CH_4, 10 mL H_2O, 0.5 h	64	0.62	0.50	(17)
10	Au-Pd colloid	50	0.5	30 mg catalyst, 3 MPa CH_4, 10 mL H_2O, 0.5 h	14	6	34.97	(18)
11	FeN_4/GN	25	0.5	30 mg catalyst, 2 MPa CH_4, 10 mL H_2O, 10 h	5	0.01	3.48	(19)
12	Fe/ZSM-5 (66)	80	5	30 mg catalyst, 3 MPa CH_4, 10 mL H_2O, 0.5 h	1.2	0.65	4.84	(20)
13	Cr_1/TiO_2	50	0.5	10 mg catalyst, 3 MPa CH_4, 9.5 mL H_2O, 1 h	8	0.38	2.84	(21)
14	Rh_1/CeO_2 NWs	50	1	10 mg catalyst, 0.5 MPa CH_4, 20 mL H_2O, 1 h	75	2.9	0.29	(22)
15	Fe-HZ5-TF	75	0.5	27 mg catalyst, 3 MPa CH_4,	5	3	39.46	(23)

				10 mL H_2O, 0.42 h				
16	Fe-HZ5-C	75	0.5	27 mg catalyst, 3 MPa CH_4, 10 mL H_2O, 0.42 h	8	1.53	12.32	(23)
17	UiO-66-H	50	0.25	10 mg catalyst, 3 MPa CH_4, 20 mL H_2O, 0.5 h	5.4	0.04	0.21	(24)

Supplementary Table S4 | Comparative experiment.

Catalyst	Productivity (mmol g_{cat}^{-1} h^{-1})				Selectivity of CH_3OH (%)	Gross productivity /mmol g_{cat}^{-1} h^{-1}
	CH_3OH	HCHO	HCOOH	CO_2		
Iron acetylacetonate	No product				--	--
$FeCl_2$	No product				--	--
$Cu(NO_3)_2$	No product				--	--
Iron acetylacetonate + $CuCl_2$ + $FeCl_2$	No product				--	--

Note: Reaction conditions: 3.5 MPa CH_4, 20 mL 0.1 M H_2O_2, 10 mg catalyst, 3 h, 80 °C.

Supplementary Table S5 | The ICP-AES and SEM-EDS results for different catalysts.

Catalyst	Fe Content (wt%)	Cu Content (wt%)
FeCu/ZSM-CI (Iron acetylacetonate)	0.67	0.50
FeCu/ZSM-CI (Ferric chloride)	0.22	0.42
Fe/ZSM-C	0.66	0
Cu/ZSM-I	0	0.51
FeCu/ZSM-CI (After the reaction)	0.64	0.48
FeCu/ZSM-CI (Iron acetylacetonate)	0.08 (SEM-EDS-mapping)	0.49 (SEM-EDS-mapping)

Note: The metal loading was confirmed by inductively coupled plasma optical emission spectrometry (ICP-AES). The reaction solution was measured by ICP. According to the data in the fifth row of the figure, ICP results show that the values of Fe and Cu after conversion are less than 0.01 mg/kg, indicating that little iron or copper cation is leaching from the catalyst.

Supplementary Table S6 | Curve-fitting parameters for Fe K-edge and Cu K-edge EXAFS in FeCu/ZSM-CI.

Sample	Shell	S_0^2	σ^2/Å^2	d^b/Å	R/Å	ΔE	CN
Fe K-edge	Fe-O_1	1.00	0.012±0.002	2.03	2.02±0.009	4.3±0.75	3.1±0.39
	Fe-O_2	1.00	0.030±0.005	3.03	3.10±0.005	4.3±0.75	2.8±0.22
Cu K-edge	Cu-O	1.00	0.004±0.001	2.12	1.95±0.006	0.45±0.61	3.3±0.18

S_0^2: amplitude reduction factor;

σ^2: Debye-Waller factor;

d: set distance;

R: fitted distance;

CN: coordination number.

[a] Date ranges: $2.5 \leq k \leq 12.3$,$1.0 \leq R \leq 3.3$ Å. The number of variable parameters is 6，4, out of a total of 11.04，24.3 independent data points. R factor for these fits are 1.8%，1.9%.

[b] The distances for Fe-O are from the crystal structure of Fe_2O_3 and Fe_4O_5; The distance of Cu-O is from the crystal structure of CuO.

It was assumed that the Debye-Waller factor is the same for all the first-shell metal pairs (Fe-O and Cu-O) to minimize the number of fitting parameters.

Supplementary References

1. Xu W, et al. Metal-oxo electronic tuning via in situ CO decoration for promoting methane conversion to oxygenates over single-atom catalysts. *Angew. Chem. Int. Ed.* **63**, e202315343, (2024).
2. Bo Wu, et al. Tandem catalysis for selective oxidation of methane to oxygenates using oxygen over PdCu/zeolite. *Angew. Chem. Int. Ed.* **61**, e202204116, (2022).
3. Wang W, et al. Selective oxidation of methane to methanol over Au/H-MOR. *J. Am. Chem. Soc.* **145**, 12928-12934, (2023).
4. Hao S, et al. Photocatalytic CH_4-to-ethanol conversion on asymmetric multishelled interfaces. *J. Am. Chem. Soc.* **146**, 25870-25877, (2024).
5. Zhang Q, et al. Efficient photocatalytic CH_4-to-ethanol conversion by limiting interfacial hydroxyl radicals using gold nanoparticles. *Angew. Chem. Int. Ed.* **64**, e202419282 (2024).
6. Xu Y, et al. Efficient methane oxidation to formaldehyde via photon-phonon cascade catalysis. *Nat. sustain.* **7**, 1171-1181, (2024).
7. Mao J, et al. Direct conversion of methane with O_2 at room temperature over edge-rich MoS_2. *Nat. Catal.* **6**, 1052-1061, (2023).
8. Qi G, et al. Au-ZSM-5 catalyses the selective oxidation of CH_4 to CH_3OH and CH_3COOH using O_2. *Nat. Catal.* **5**, 45-54, (2022).
9. Hao S, et al. Switching photocatalytic methane oxidation toward ethanol by tuning spin states. *Angew. Chem. Int. Ed.* **64**, e202510241, (2025).
10. Xue F, et al. Selective light-driven methane oxidation to ethanol. *Nat. Commun.* **15**, 10451, (2024).
11. Yu B, et al. Silver and copper dual single atoms boosting direct oxidation of methane to methanol via synergistic catalysis. *Adv. Sci.* **10**, 2302143, (2023).
12. Yu T, et al. Highly selective oxidation of methane into methanol over Cu-promoted monomeric Fe/ZSM-5. *ACS Catal.* **11**, 6684-6691, (2021).
13. Tang X, et al. Direct oxidation of methane to oxygenates on supported single Cu atom catalyst. *Appl. Catal. B Environ.* **285**, 119827, (2021).
14. Hammond C, et al. Direct catalytic conversion of methane to methanol in an aqueous

medium by using copper-promoted Fe-ZSM-5. *Angew. Chem. Int. Ed.* **51**, 5129-5133, (2012).

15. Ab Rahim MH, et al. Oxidation of methane to methanol with hydrogen peroxide using supported gold-palladium alloy nanoparticles. *Angew. Chem. Int. Ed.* **52**, 1280-1284, (2012).
16. Huang W, et al. Low-temperature transformation of methane to methanol on Pd_1O_4 single sites anchored on the internal surface of microporous silicate. *Angew. Chem. Int. Ed.* **55**, 13441-13445, (2016).
17. Kwon Y, et al. Selective activation of methane on single-atom catalyst of rhodium dispersed on zirconia for direct conversion. *J. Am. Chem. Soc.* **139**, 17694-17699, (2017).
18. Jin Z, et al. Hydrophobic zeolite modification for in situ peroxide formation in methane oxidation to methanol. *Science* **367**, 193-197, (2020).
19. Cui X, et al. Room-temperature methane conversion by graphene-confined single iron atoms. *Chem* **4**, 1902-1910, (2018).
20. Zhu K, et al. Highly efficient conversion of methane to formic acid under mild conditions at ZSM-5-confined Fe-sites. *Nano Energy* **82**, 105718, (2021).
21. Shen Q, et al. Single chromium atoms supported on titanium dioxide nanoparticles for synergic catalytic methane conversion under mild conditions. *Angew. Chem. Int. Ed.* **59**, 1216-1219, (2019).
22. Bai S, et al. High-efficiency direct methane conversion to oxygenates on a cerium dioxide nanowires supported rhodium single-atom catalyst. *Nat. Commun.* **11**, 954, (2020).
23. Cheng Q, et al. Maximizing active Fe species in ZSM-5 zeolite using organic-template-free synthesis for efficient selective methane oxidation. *J. Am. Chem. Soc.* **145**, 5888-5898, (2023).
24. Fang G, et al. Zirconium-oxo nodes of MOFs with tunable electronic properties provide effective •OH species for enhanced methane hydroxylation. *Angew. Chem. Int. Ed.* 61, e202205077, (2022).
